# Meeting of the American Medical Association

**Published:** 1858-08

**Authors:** 


*Meeting of the American Medical Association at Washington, May,* 1858.—A Delegate from Massachusetts in a recent number of the Boston Medical and Surgical Journal, makes the following remarks: "Unless some means can be taken, the association will become simply an arena for the display of private pique or public denunciation, or for arguments on constitutional questions." In reference to the recent meeting, aside from the pleasure of meeting former acquaintances and friends, and of partaking of the hospitalities of the committee of arrangements and citizens of Washington, how much was there of interest pertaining to the legitimate business of the association? Much time was spent discussing the subject of discipline, and considerable time in discussing and reconsidering the recommendation of Dr. Bailey as Inspector of Drugs, a subject which should never have been brought before the association. Whilst the reports of several committees were crushed out, the authors not having the poor privilege of presenting them.

Many of my associates think the discussion on the subject of discipline was called for, and will prove useful; my own view is, that matters of discipline should be left to the local societies, and should never come before the association except on appeal, and should then be referred to a committee to obtain the facts and report at a special meeting of the association. It is certainly poor encouragement to spend much time and labor in preparing a report, with no certainty that when prepared the author will be permitted to present it to the association.

Much of the difficulty of the recent meeting, it is true, might have been prevented by a strict adherence to the established regulations of the association. There are, however, certain reforms, the necessity for which seem so self-evident, that no one could object:

1. The election of president should be at the close and not at the opening of the meeting, to take his seat the next year, he would thus have time to prepare himself for the duties, and the members would have an opportunity of comparing views before being called upon to vote.

2. The practice of always selecting the president from the place of meeting should be abandoned.

3. Every committee having a report to make, should have an opportunity of presenting it, and if desired, of briefly stating the contents.

4. Matters of discipline should never come before the association except on appeal from the local society, and should then be referred to a committee to report at a special meeting of the association.

5. The regular order of business should be strictly adhered to, and no miscellaneous business be permitted to interfere with the reports from the several committees. If there is miscellaneous business that cannot be deferred, let a specific time, at some extra hour, be assigned for its consideration so that those who did not wish to listen should not be compelled to attend; whilst I would give every one who desired it an opportunity of displaying their eloquence, I would not compel unwilling listeners to attend under the penalty of missing the regular business, or consume the time which should be devoted to the regular business.

No change in the constitution is needed to effect these reforms. A vote of two-thirds to change the time of electing the president, and a strict adherence to the established regulations of the association, is all that is required.

A DELEGATE FROM N. Y.

*Philadelphia Alms-House or Blockley Hospital.*—"We have room merely to announce that Dr. R. K. Smith has been reëlected Chief Resident Physician to the Philadelphia Hospital, Blockley—thus effectually redeeming that institution from the disgraceful position which it has for some time occupied."

We copy the above announcement from the Medical and Surgical Reporter for July, and join with the editor most cordially, in rejoicing that Blockley Hospital is at last redeemed from quackery, or what is worse, renegade quackery. In our July number, 1857, we merely made the announcement that Dr. Jas. McClintock was appointed to a post so honorable and responsible as that of Chief Resident at Blockley, stating the probable occasion of his repentance and return to the legitimate practice, and nothing more. During the war which was waged against Drs. Reese and Bryan we said nothing, willing to think that the kindly feelings of the man overruled what was certainly the duty of the Vice-president of the American Medical Association, and perfectly satisfied to allow the matter to be settled by that body. Though we certainly did not approve of the venomous mode of defence which Dr. Reese adopted, and would have been much better pleased with an explanation not followed up by personal attacks upon others, yet we could not but approve of his course before the American Medical Association, and thought that the action of that body ought to be satisfactory to all parties. Dr. Reese has been an ardent and successful worker in the profession, which fact those who denounced him so violently did not appear to recollect, and it was certainly due to him to believe that his heart was stronger than his head.

We had intended to let the matter pass entirely, but have been led into these few remarks by the unexpected announcement which heads the paragraph. The governors of institutions like the Philadelphia Almshouse, will find that it is impossible to do anything so directly in the face of the profession, as the appointment of a quack, or a reformed quack, to an important post. Let him live in the practice of his profession as before; we do not wish to crush a fellow-man, especially when he appears before us as a penitent, no matter how shallow we may think his repentance; but do not let us support him for a responsible and lucrative position, in justice to our brethren who have been firm and steadfast. We know that it is hard to live in a large city by the legitimate practice of medicine, but the way of the transgressor is harder. The consciousness of an unblemished honor, the respect of good men, and a love for true science, are necessary to support the physician through the trials and sorrows which surround him.

